# Supplementary material for: The Safety of Negative-Pressure Wound Therapy in Melanoma and Sarcoma Patients: A Systematic Review
Source: J Clin Med. 2025 Oct 5;14(19):7044. doi: 10.3390/jcm14197044 (PMC12524936; doi:10.3390/jcm14197044)
Supplement: Supplementary file 1 [file jcm-14-07044-s001.zip › jcm-3860740-supplementary.pdf]

Supplementary Table S1. Main aim of the included studies [10–26].

Author	Aim of the study
Loos B [10]	To evaluate the efficacy of NPWT with staged debridement in the reintegration or integration of alloplastic meshes.
Senchenkov, A. [11]	To evaluate the outcomes of split-thickness skin grafts following oncologic resections in patients with radiotreated sarcomas.
Heller L [12]	To report the use of NPWT applied over brachytherapy catheters, followed by delayed flap reconstruction, in 3 patients with soft tissue sarcoma treated postoperatively with brachytherapy
Oh B. H [22]	To evaluate the effectiveness of NPWT in the healing of large size defect after wide excision of Acral Melanoma compared to secondary healing
Agostini T [13]	To report a single center experience in the management of large size DFSP
Seo J [24]	To evaluate the efficacy and adverse effects of a combination treatment involving NPWT and punch skin grafting.
Bedi M [25]	To evaluate the effects of NPWT of the development of wound complication after lower extremity sarcoma resection and the risk of local relapse.
Wu M [14]	To explore the efficacy of keystone flap combined with the VAC therapy in the repair of sacrococcygeal wounds.
Jørgensen, M.G. [23]	To examine the effectiveness of a portable iNPWT in preventing seroma, SSI, and lymphedema after ILND in patients with malignant melanoma.
Baysal Ö [15]	To investigate the risk factors identified in literature that have been associated with prolonged NPWT
Lembo F [16]	To report a single center experience with the use of NPWT in sarcoma patient
Miura T [17]	To evaluate the feasibility of a new method of internal and external negative pressure to treat lymphocutaneous fistula after lymph node dissection for melanoma.
Korovin, S [18]	To estimate the engraftments of grafts using VAC and explore factors influencing this process
Gjorup G.A. [19]	To report the outcome of a secondary closure healing after wide excision of melanoma.
Fourman MS. [20]	To evaluate the risk of local recurrence of VAC temporization after sarcoma excision
Chen Y [21]	To evaluate the efficacy and safety of NPWT after resection of extremities STS
Shields D. W. [26]	To compare the postoperative infection rate between NPWT and conventional dressings for closed incisions following STS surgery. Secondary objectives were to compare rates of adverse wound events and functional scores.
